# CPExtract, a Software Tool for the Automated Tracer-Based Pathway Specific Screening of Secondary Metabolites in LC-HRMS Data

**DOI:** 10.1021/acs.analchem.1c04530

**Published:** 2022-02-15

**Authors:** Bernhard Seidl, Rainer Schuhmacher, Christoph Bueschl

**Affiliations:** University of Natural Resources and Life Sciences, Vienna, Department of Agrobiotechnology (IFA-Tulln), Institute of Bioanalytics and Agro-Metabolomics, Konrad-Lorenz-Straße 20, 3430 Tulln, Austria

## Abstract

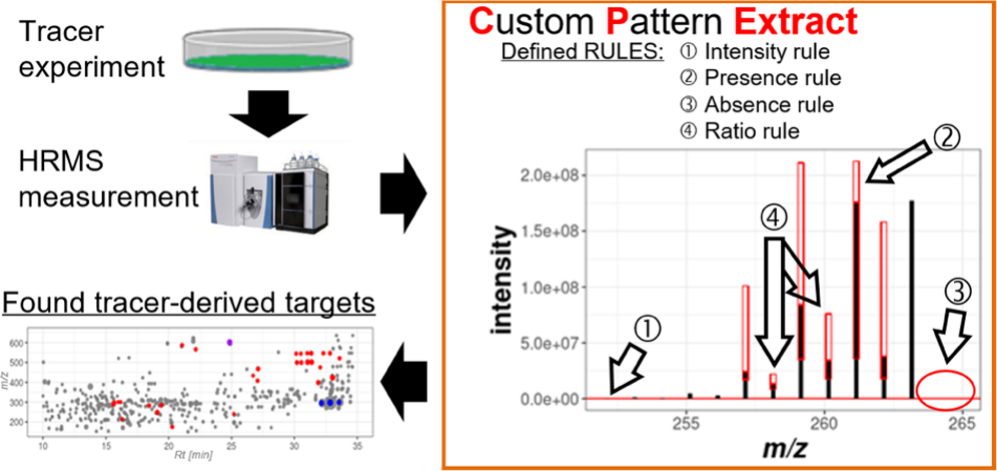

The use of stable isotopically labeled tracers is a long-proven way of specifically detecting and tracking derived metabolites through a metabolic network of interest. While the recently developed stable isotope-assisted methods and associated, supporting data analysis tools have greatly improved untargeted metabolomics approaches, no software tool is currently available that allows us to automatically and flexibly search liquid chromatography coupled with high-resolution mass spectrometry (LC-HRMS) chromatograms for user-definable isotopolog patterns expected for the metabolism of labeled tracer substances. Here, we present Custom Pattern Extract (CPExtract), a versatile software tool that allows for the first time the high-throughput search for user-defined isotopolog patterns in LC-HRMS data. The patterns can be specified via a set of rules including the presence or absence of certain isotopologs, their relative intensity ratios as well as chromatographic coelution. Each isotopolog pattern satisfying the respective rules is verified on an MS scan level and also in the chromatographic domain. The CPExtract algorithm allows the use of both labeled tracer compounds in nonlabeled biological samples as well as a reversed tracer approach, employing nonlabeled tracer compounds along with globally labeled biological samples. In a proof-of-concept study, we searched for metabolites specifically arising from the malonate pathway of the filamentous fungi *Fusarium graminearum* and *Trichoderma reesei*. 1,2,3-^13^C_3_-malonic acid diethyl ester and native malonic acid monomethyl ester were used as tracers. We were able to reliably detect expected fatty acids and known polyketides. In addition, up to 46 and 270 further, unknown metabolites presumably including novel polyketides were detected in the *F. graminearum* and *T. reesei* culture samples, respectively, all of which exhibited the user-predicted isotopolog patterns originating from the malonate tracer incorporation. The software can be used for every conceivable tracer approach. Furthermore, the rule sets can be easily adapted or extended if necessary. CPExtract is available free of charge for noncommercial use at https://metabolomics-ifa.boku.ac.at/CPExtract.

## 1 Introduction

Untargeted metabolomics aims at the unbiased detection of all metabolites produced by a biological system that is covered by the respective analytical method used. Typically both qualitative and quantitative differences in metabolites resulting from natural fluctuations, genetic modifications (e.g., wild-type (WT) versus knock-out), or experimental biotic or abiotic stress factors (e.g., untreated versus treated in any form) are then examined.^1^ Nowadays, besides nuclear magnetic resonance (NMR) spectroscopy, liquid chromatography coupled with high-resolution mass spectrometry (LC-HRMS) is the most commonly used analytical technique to cope with this task. LC-HRMS allows the detection of a large number of different metabolites in the samples. However, usually, only a few of these can be identified by comparison with reference standards.^2^ Moreover, it is often not even possible to reliably differentiate between signals derived from contaminants or artifacts and those of truly biological metabolites.^3^ To encounter this problem, stable isotopically labeled samples, biological material, or tracer molecules can be employed. The ingenious basic principle here is that labeling is incognito for the organism, i.e., labeled substances are metabolized almost the same way as unlabeled substances in biochemical reactions, but the label can be easily traced in analytical measurements.^4,5^ Stable isotope-assisted (SIA) workflows were used as early as the 1950s in comprehensive studies of metabolic pathways, where the progression of biochemical signaling pathways and metabolic intermediates were investigated by tracking changes in the isotopic composition of metabolites of the metabolic pathway under study over time. This made it possible to study metabolic pathways and networks or to investigate the metabolic fate of substances (e.g., drugs or toxins) into intermediate or end products. Typically, ^13^C, ^15^N, or ^2^H isotopes are used for tracking derived metabolites through a given metabolic network of interest.^5−9^

Additionally, the labeling information can also be used for improved unknown compound annotation, e.g., by means of interpretation of SIA tandem mass spectrometry data.^10^

Meanwhile, software-supported approaches for the untargeted search for tracer-derived compounds are also commonly used in metabolomics, where in contrast to the more specific biosynthetic pathway studies, a snapshot of the entire metabolic state that can be captured by the method in use is analyzed.^1^ In the latter case, the artificial isotope patterns caused by the metabolism of the labeled tracer are used to reliably track all tracer-derived metabolites that contain the entire tracer or parts thereof.

However, especially in untargeted SIA metabolomics studies, the huge amounts of raw LC-HRMS data do not allow the comprehensive, manual evaluation anymore. Therefore, a variety of (semi)automated SIA methods and data evaluation tools have recently been developed which greatly help to improve untargeted metabolomics approaches. Examples of such tools are MetExtract II,^11^ X13CMS,^12^ HiTIME,^13^ geoRge,^14^ ALLocator,^15^ NTFD,^16^ or Compound Discoverer 3× (Thermo Scientific). These software tools aim at the unbiased detection of all labeled metabolites and require a high degree of isotopic enrichment resulting in completely separated isotopolog patterns of the unlabeled and labeled metabolites, respectively. Each of these tools has been developed for particular, rigidly predefined isotope patterns or is based on direct comparison of native and labeling-derived isotope signatures and is therefore tailored to its respective specific application. For example, MetExtract II^11^ and HiTIME^13^ both require LC-HRMS data sets consisting of native and uniformly ^13^C-labeled compounds and thus detect either such labeled metabolites or their biotransformation products (e.g., mycotoxins, endogenous metabolites, or drugs). On the other hand, X13CMS,^12^ geoRge,^14^ or NTFD^16^ aim at the untargeted search for the totality of all existing isotope-enriched metabolites that potentially have alterations in their isotope patterns after treatment with an isotopically labeled precursor (such as U-^13^C_6_ glucose) in comparison to the same samples treated with the identical but nonlabeled precursor. These tools require the separate measurement of unlabeled and labeled samples. The assignment of labeled constituents is then based on the differential comparison of isotopolog patterns between labeled and unlabeled samples, and thus indirectly relies on correction for natural isotopic abundance to find all labeled compounds in the biological sample under investigation.

To the best of the authors’ knowledge, however, there is no software tool available to date that allows the user to freely and flexibly specify a custom isotopolog pattern, which is then searched for in the LC-HRMS data set. Here, we present Custom Pattern Extract (CPExtract) that allows the user to specify custom isotopolog patterns together with preset relative intensity ranges to be searched for, thereby enabling specific filtering for metabolite ions of interest that agree with the desired and diagnostic isotopolog pattern.

In contrast to the aforementioned software, CPExtract does not have any rigid specifications but allows the user to define the isotopolog patterns to be searched for and the corresponding criteria freely via various rules. This allows, for example, the highly targeted search for a very specific isotopolog pattern that can typically be expected to occur after multiple, iterative tracer incorporation during the biosynthesis of certain classes of specialized metabolites, as in the application presented here.

## 2 Methods

### 2.1 CPExtract Application Example

The application of CPExtract is presented by searching specifically for metabolites of the malonate pathway, namely, fatty acids, polyketides, and possible hybrid metabolites thereof (e.g., nonribosomal peptide-polyketides (NRP-PKs) or prenylated polyketides) in culture samples of the filamentous fungi *Fusarium graminearum* and *Trichoderma reesei*.

Two complementary tracer approaches were used. First, 1,2,3-^13^C_3_-malonic acid diethyl ester was used as a tracer substance. This approach will henceforth be referred to as the standard tracer approach since the tracer was isotopically labeled (^13^C) and the cultivation was carried out with native glucose as the sole carbon source in the growth medium.

The second tracer substance was malonic acid monomethyl ester. Since this substance is not readily available in a ^13^C isotopically labeled form, a complementary approach was chosen, which will henceforth be referred to as the reversed tracer approach. Here, the native form of malonic acid monomethyl ester was used as a tracer, while the only other available carbon source for the fungus was U-^13^C_6_-labeled glucose. Detailed information about the cultivation workflow can be found in the Supporting Information.

### 2.2 CPExtract Algorithm

**Figure 1:**
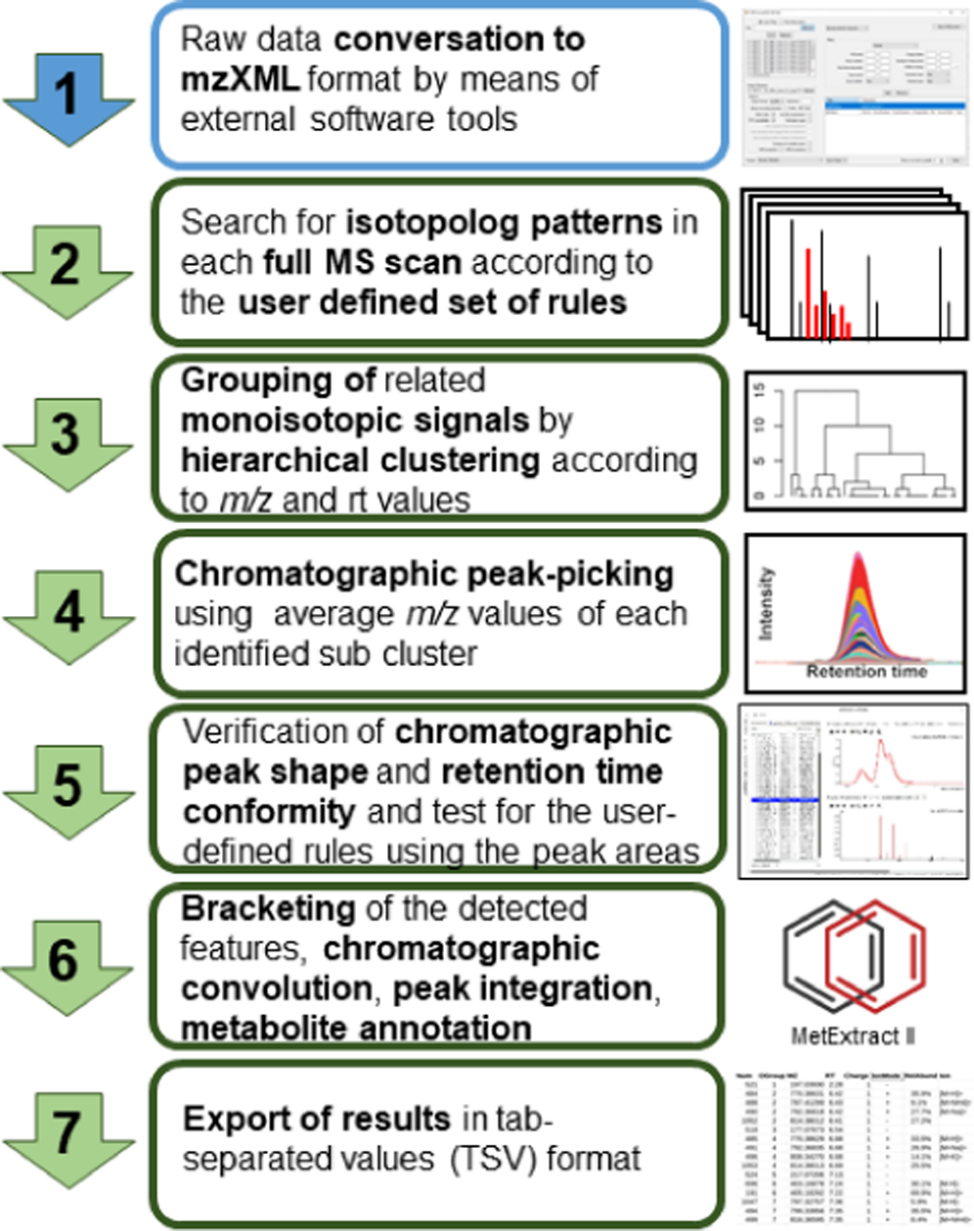
Schematic overview chart of the data processing workflow and CPExtract algorithm.

The automated CPExtract workflow is based on MetExtract II software, which originally has been designed to detect pairs of native and uniformly ^13^C-labeled metabolite ions. CPExtract replaces this fixed and rigid definition with a set of freely definable rules by which the isotope patterns to be searched for can be arbitrarily defined by the user. The individual rule set is defined via a Python vector of objects where each is an instance of one of the currently available “Rule” objects, which include the presence or absence of certain isotopologs as well as abundance or area-ratios between different isotopologs. Further custom user-defined rules can be easily added by defining additional subclasses. Automatic data processing then filters for all signals with isotope patterns that correspond to the defined set of rules. A schematic representation of the data processing workflow and algorithm is shown in Figure 1.

The individual steps of the processing workflow and CPExtract algorithms are:

1. To be processed, the manufacturer-specific raw data chromatograms must be converted into the mzXML format with, for e.g., ProteoWizard MSConvert.^17^
2. Data processing in CPExtract starts at the MS scan level, whereby each individual *m*/*z* signal in a full scan mass spectrum is initially considered to represent the principal isotopolog of a putative metabolite ion of interest (henceforth termed X). This assumption is then verified by checking the user-defined set of rules. Currently, the following distinct rules are available: the “PresenceRule” (a particular isotopolog must be present and be within expected ratios relative to other isotopologs) and “AbsenceRule” (a specified isotopolog must not be present). For these two rules, a “minIntensity” parameter, which can be used to define a minimum signal intensity that the isotopolog must have, and a “verifyChromPeaksSimilarity” parameter, which specifies the chromatographic peaks of the isotopologs, must be present and coelute. Furthermore, the “RatioRule” (checks whether an intensity ratio of two isotopologs or even two groups of several summed up isotopolog intensities corresponds to a certain defined ratio or checks if both ratios are within a defined range), the “AnyIntensityRule” and the “AllIntensityRule” (either any or all specified isotopologs must exceed a certain intensity threshold) are available. Moreover, users can easily implement their own rules if required. It is then checked if the isotopologs defined in the rule set relative to the assumed X isotopolog are valid (e.g., if certain isotopologs are present or absent). Only if X and all its putatively related isotopolog peaks meet the specified rules, X is used for subsequent data processing.
3. All MS signals (i.e., Xs) that fulfilled the rules in the previous step are then clustered using hierarchical clustering. Subclusters of the thereby generated dendrogram within a low mass-deviation window (scan-to-scan variability) are kept. This dendrogram cutting step also takes the acquisition time (scan number according to the retention time) of the MS scans into consideration so that noncoeluting isomers end up in different subclusters.
4. Data processing then continues on the chromatographic level. Therefore, an average *m*/*z* value is calculated for X of each subcluster present from the previous step, and peak picking is carried out using the continuous wavelet transform (CWT)-based centWave algorithm.^18^
5. Only those isotope pattern candidates (or Xs) identified in the previous steps, whose chromatographic retention and peak shape show sufficient agreement with the user’s rules, are ultimately accepted as hits. For this, all chromatographic peaks of the isotopologs must show perfect chromatographic coelution, which is tested via the Pearson correlation coefficient. Additionally also the isotopolog ratios based on the chromatographic peak areas are again checked and must meet the user’s rule criteria.
6. The remaining steps of CPExtract are the same as those implemented in MetExtract II,^11^ namely, bracketing of the detected features across all samples, chromatographic convolution (i.e., grouping and annotation of metabolite ions from the same metabolite), and peak area reintegration.
7. Results of the automated data processing are provided in the form of a tab-separated value (TSV) file containing a data matrix with all detected principal isotopologs (Xs). The results can also be visualized and checked in the software’s graphical user interface, whereby both the extracted ion chromatograms and the spectra are illustrated.

### 2.3 Fungal Strains and Cultivation Conditions

*F. graminearum* PH-I (wild-type) obtained from Gerhard Adam, BOKU, Institute for Microbial Genetics (IMiG), and three different *T. reesei* strains, namely, QM6aΔ*tmus53* (wild-type),^19^ QM6a Δ*tmus*53Δ*sor1* (a knock-out mutant lacking the gene encoding the SOR1 polyketide synthase, the first enzyme in the sorbicillinoid biosynthetic pathway),^20^ and QM6a Δ*tmus53ReYpr*1 (a yellow pigment regulator 1 transcription factor overexpressing strain)^21^ were used for the preparation of the culture medium supernatant and mycelium samples. To obtain the data sets, the samples were measured by LC-HRMS. Detailed information about the SIA-tracer workflow, cultivation, sample preparation, and measurement are given in the Supporting Information.

### 2.4 Conversion of LC-HRMS Raw Data Files

The raw data files were converted into the mzXML format using the ProteoWizard MSConvert Software^17^ version 3.0.19210 (settings: mzXML output format, 32-bit binary encoding precision, enabled index writing, enabled TPP compatibility, disabled zlib and gzip compression, peak picking using vendor algorithm).

### 2.5 CPExtract Rules and Parameter Settings for Data Processing

#### 2.5.1 Processing of LC-HRMS Data Generated Using the Standard Tracer Approach

The following set of rules was used to detect isotopolog patterns of fully ^12^C metabolites that incorporate several ^13^C_2_ units derived from the ^13^C-labeled malonic acid diethyl ester tracer as expected from the standard tracer approach:

X represents the native, monoisotopic form of the metabolite ions and consists only of ^12^C isotopes for each carbon atom, and X_+υ_ indicates that υ ^12^C atoms are exchanged for ^13^C atoms in that particular isotopolog relative to X.

- X must have a signal abundance of at least 1E5 counts. X_+2_ and X_+4_ must have a signal intensity of at least 5E4 counts. This is used to consider only signals with a sufficient signal intensity and, for e.g., mask out signal noise (part of PresenceRule).
- As X already represents the monoisotopic^12^C isotope form of the metabolite, the signals for putative ^12^C_–1_ and ^12^C_–2_ isotopologs, therefore, must not be present, otherwise, they would be false positives. To avoid false-negative results, signals with an intensity value of a maximum of 5% of X are accepted as putative noise signal for both isotopologs without a rule violation (AbsenceRule).
- The metabolite ion containing one and two ^13^C-malonate tracer-derived extension units X_+2_ and X_+4_ must be present and their abundance has to be within the range of 10–300% of X and X_+2_, respectively. (RatioRule)
- Moreover, the metabolite ions containing one or two partial isotopically labeled extension units (respectively, one ^13^C and one ^12^C) X_+1_ and X_+3_ must also be present and must have an abundance of 10–200% relative to X and X_+2_ as well as X_+2_ and X_+4_, respectively (RatioRule).
- The ratio of the abundance of X totaled with the abundance of X_+2_ to the abundance of X_+1_ must be the same as of the abundance ratio of X_+2_ totaled with the abundance of X_+4_ to the abundance of X_+3_ with a maximum permissible relative deviation of these ratios of ±10%, respectively (i.e., (X + X_+2_)/X_+1_ ≈ (X_+2_ + X_+4_)/X_+3_). This rule takes advantage of the natural isotope distribution and the isotopic impurity of the tracer substance to remove false positives (RatioRule).
- The isotopologs X, X_+1_, X_+2_, X_+3_, and X_+4_ must be present as coeluting chromatographic peaks (Pearson correlation ≥ 0.85) (part of the first PresenceRule).

#### 2.5.2 Processing of LC-HRMS Data Generated Using the Reversed Tracer Approach

To detect isotopolog patterns of fully ^13^C-labeled metabolites that incorporate one or more ^12^C_2_ units derived from the native malonic acid monomethyl ester, slightly modified rules compared to the standard tracer approach were used.

In the case of the reversed tracer approach, X represents the uniformly ^13^C-labeled form of the metabolite ions and consists only of ^13^C isotopes for each carbon atom and X_–υ_ indicates that υ ^13^C atoms are exchanged for ^12^C atoms.

- X must have a signal abundance of at least 1E5 counts. X_–2_, X_–4_, and X_–6_ must have a signal intensity of at least 5E4 counts (PresenceRule).
- Since here X already represents the fully labeled ^13^C isotope form of the metabolite, the signals for putative ^13^C_+1_ and ^13^C_+2_ isotopologs should not be present. Signals up to 5% of X were tolerated (AbsenceRule).
- The metabolite ion containing one, two, and three ^12^C-malonate tracer-derived extension units X_–2_, X_–4_, and X_–6_ must be present and their abundance has to be in the range of 10–300% of X, X_–2_, and X_–4_, respectively (RatioRule).
- Moreover, the metabolite ions containing one or two extension units with one ^12^C and one ^13^C (i.e., X_–1_, X_–3_, and X_–5_) must be present and must have an abundance of 10 to 200% relative to X and X_–2_, X_–2_ and X_–4_, and X_–4_ and X_–6_, respectively (RatioRule).
- The ratio of the abundance of X totaled with the abundance of X_–2_ to the abundance of X_–1_ must be the same as of the abundance ratio of X_–2_ totaled with the abundance of X_–4_ to the abundance of X_–3_ as well as the abundance ratio of X_–4_ totaled with the abundance of X_–6_ to the abundance of X_–5_ with a maximum permissible deviation of these ratios of ±10%, respectively (i.e., (X + X_–2_)/X_–1_ ≈ (X_–2_ + X_–4_)/X_–3_ ≈ (X_–4_ + X_–6_)/X_–5_) (RatioRule).
- All isotopologs defined via “PresenceRule” instructions (X, X_–1_, X_–2_, X_–3_, X_–4_, X_–5_, and X_–6_) had to be present as coeluting chromatographic peaks and must show a highly similar peak shape (Pearson correlation ≥ 0.85) (part of the first PresenceRule).

#### 2.5.3 General Processing Parameters

The further data processing parameter settings of CPExtract were: intra-scan-mass-accuracy: ±5 ppm, inter-scan-mass-deviation: ±8 ppm, EIC-extraction window: ±5 ppm, retention-time-window: 3–36 min, minimum/maximum chromatographic peak width: 5–25 s, minimum Pearson correlation for coeluting chromatographic peaks: 0.85, maximum allowed retention-time-deviation for bracketing of multiple measurements: 0.1 min, and maximum allowed *m*/*z* deviation for bracketing: 5 ppm.

## 3 Results and Discussion

A novel software tool, named CPExtract, for the comprehensive and automated search for isotope patterns in LC-HRMS data that can be freely defined by the user is presented. Other than the existing software tools, it provides the user with an automated framework to mine the LC-HRMS data for certain characteristic isotopolog patterns via a set of predefined and extensible rules. Only isotopolog patterns obeying these rules are reported. Moreover, the large flexibility in defining the target patterns enables us to use the CPExtract algorithm in two complementary ways, a standard tracer-, as well as a reversed tracer approach. As a proof-of-concept, CPExtract software was used to search specifically for fungal metabolites of the malonate pathway, namely, fatty acids and polyketides as well as their derivatives like, for e.g., nonribosomal peptide-polyketide (NRP-PK) or prenyl-polyketide hybrids in LC-HRMS data.

### 3.1 Expected Theoretical Isotope Patterns

#### 3.1.1 Standard Tracer Approach

**Figure 2:**
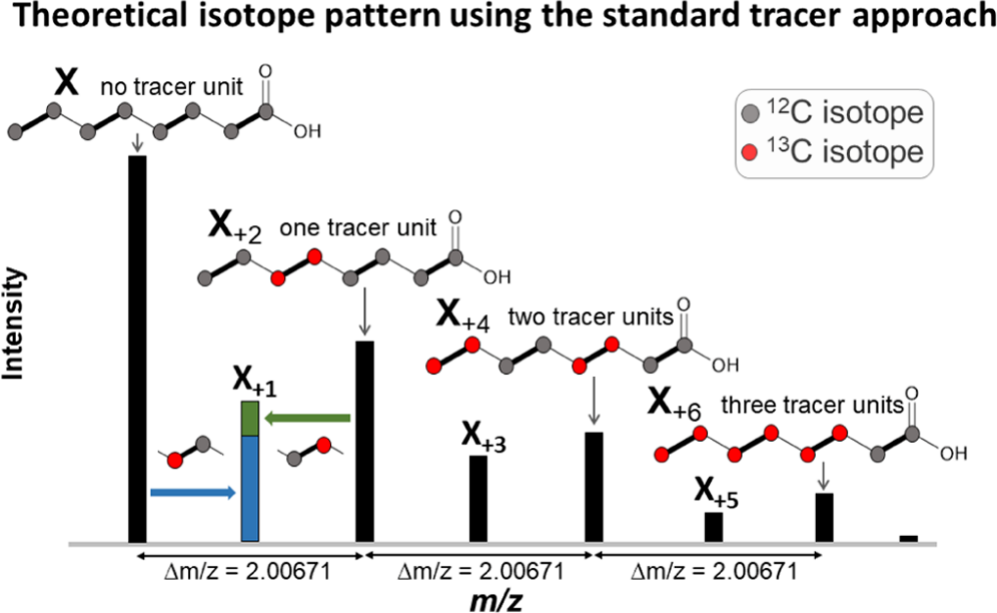
Theoretical isotope pattern of caprylic acid, which can be expected as a result of the incorporation of C_2_-extension units derived from both, native malonate (synthesized by the fungus itself) and stable isotopically labeled malonate (tracer added to the medium) into the growing hydrocarbon chain.

Figure 2 shows a typical theoretical isotopolog pattern of a substance that is to be expected for the standard tracer approach, due to the accidental incorporation of both added (^13^C-) tracer-derived and native fungus-produced C_2_ building blocks into its carbon skeleton. Such a pattern can be expected when the bioavailability and incorporation rate of tracer molecules is small in relation to the amount of malonyl-CoA units, endogenously formed from native glucose and used by the fungus itself for biosynthesis.

A staircase-shaped isotope pattern is characteristic of continuously produced metabolites, corresponding to the decreasing probability that an increasing number of tracer-derived C_2_ units become incorporated into the synthesized metabolite.

In addition, also the X_+1_, X_+3_, and X_+5_ isotopologs are of high diagnostic value. They result only from the proportion of non-monoisotopic tracer-derived extension units incorporated into the metabolites that occur due to the natural abundance of ^13^C isotopes in the native carbon source and the ^12^C isotopic impurity of the ^13^C-labeled tracer compound used. Therefore, their signal intensities must be smaller than the mean value of the two neighboring isotopologs with an even-numbered mass increment (e.g., X_+1_ < (X + X_+2_)/2). Furthermore, since the natural isotope abundance and isotopic purity of the tracer are constant, the resulting intensity ratio (e.g., (X + X_+2_)/X_+1_) must be almost the same for all analogous isotopolog pairs (e.g., (X_+2_ + X_+4_)/X_+3_). The intensity ratio of odd-numbered isotopologs to their even-numbered neighbors is independent of the number of tracer-derived extension units incorporated into the particular metabolite ion, making them ideal for highly specific filtering.

#### 3.1.2 Reversed Tracer Approach

**Figure 3:**
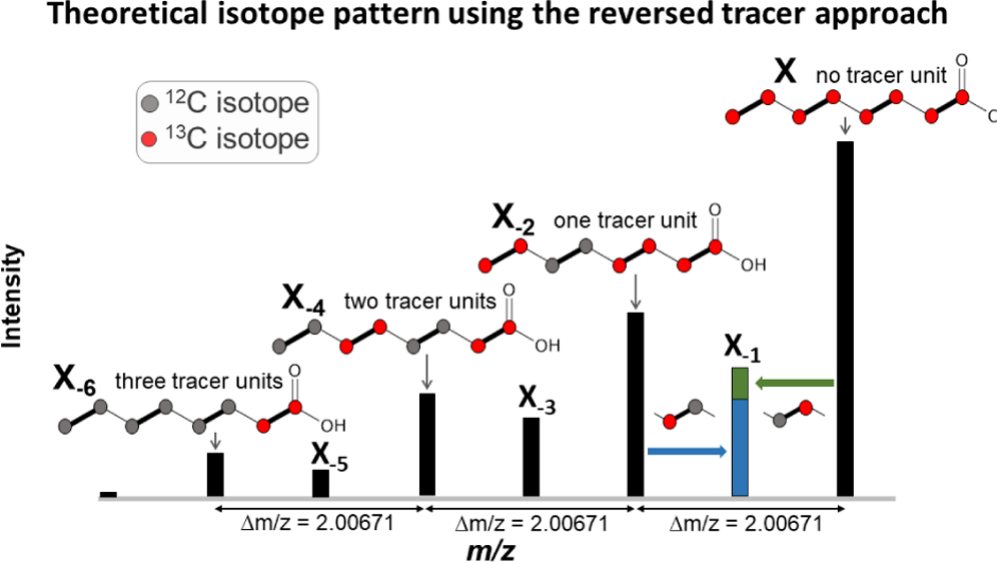
Theoretical isotope pattern of caprylic acid, which can be expected as a result of the incorporation of C_2_ units derived from both, ^13^C malonate (synthesized by the fungus itself as grown on U-^13^C_6_-glucose as the sole carbon source) and native malonate (tracer added to the medium) into the growing hydrocarbon chain.

For experiments that require the use of compounds that are not available in the ^13^C-labeled form or would be too expensive, a reversed approach provides a solution. This approach combines global ^13^C labeling of the biological system with the use of a native tracer compound (such as malonic acid monomethyl ester). The thereby generated isotope patterns are a mirror image inversion of the patterns expected from the standard tracer approach (Figure 3).

Due to the high flexibility in defining rules for the isotope patterns to be searched for, CPExtract software works with both tracer approaches.

#### 3.1.3 Exemplification of Isotope Patterns and Tolerance Intervals for Oleic Acid

**Figure 4:**
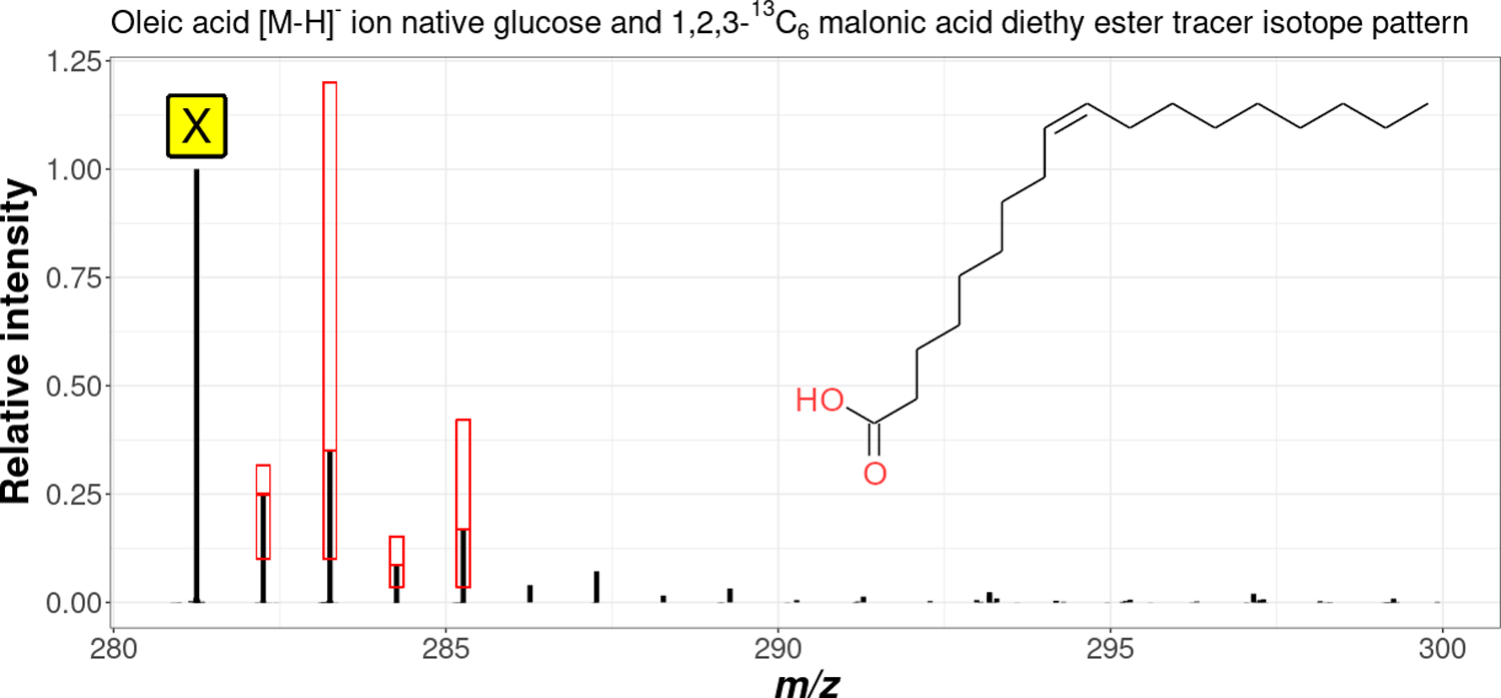
Measured spectrum of the [M – H]^−^ ion of oleic acid in the mycelium extract samples of *F. graminearum* using the standard tracer approach. X denotes the predominant, monoisotopic, native deprotonated molecule. Also shown in red boxes are the isotopologs defined in the rules that must be present and their permitted intensity ranges.

**Figure 5:**
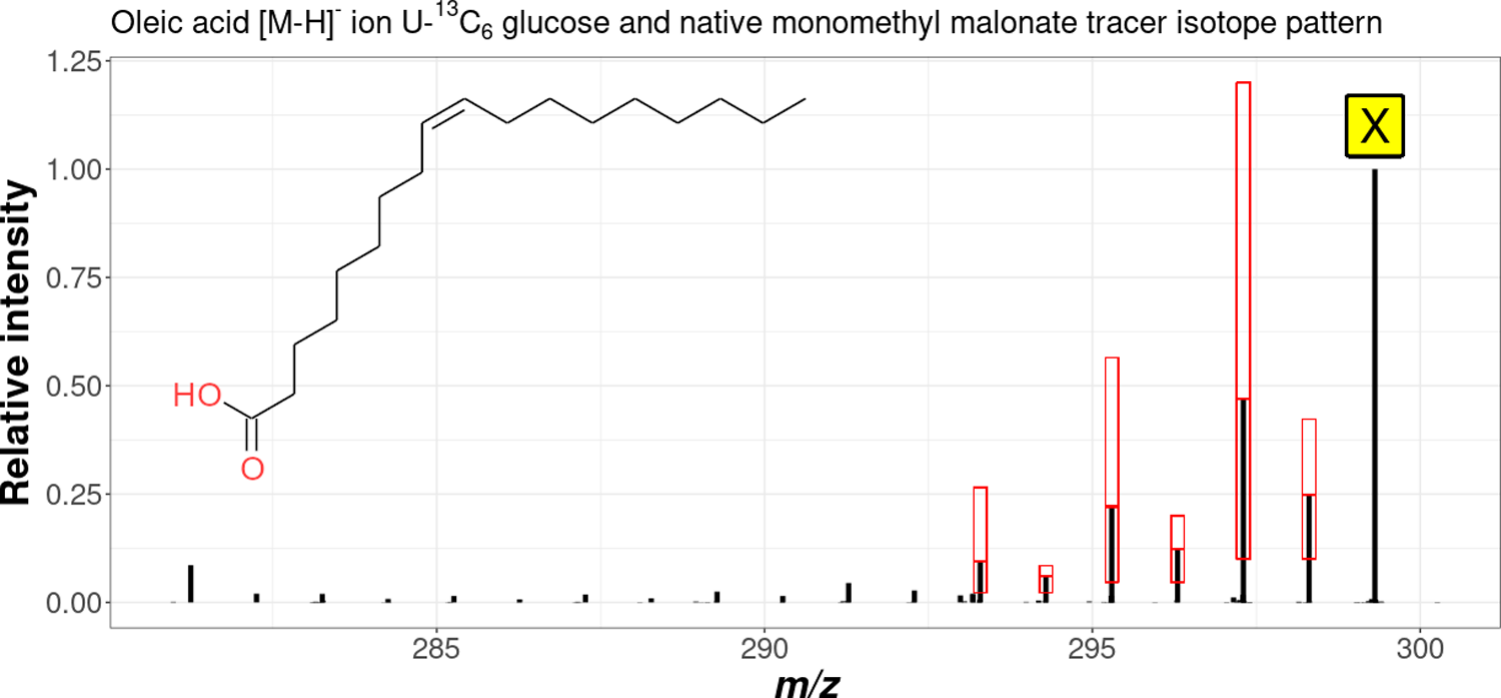
Measured spectrum of the [M – H]^−^ ion of oleic acid in the mycelium extract samples of *F. graminearum* using the reversed tracer approach. X denotes the predominant, monoisotopic fully ^13^C-labeled deprotonated molecule. Also shown in red boxes are the isotopologs defined in the rules that must be present and their permitted intensity ranges.

Figures 4 and 5 show the spectra of oleic acid experimentally measured in *F. graminearum* mycelium extract samples together with the identified monoisotopic isotopolog (X) and the respective tracer-derived isotopologs exemplarily for the standard tracer- and reversed tracer approach, respectively. For the *T. reesei* mycelium extract, similar patterns were observed (data not shown). In both illustrations, the red boxes show the required isotopologs as well as their permissible intensity ranges as defined in the respective rule set for the standard- and the reversed tracer approach.

As can be seen in Figures 4 and 5, the rules selected are suitable for the detection of the isotope patterns in the measurement data caused by the random incorporation of tracer-derived C_2_ extension units. Due to the rapidly decreasing signal intensity of isotopologs with an increasing number of tracer-derived extension units, not all theoretically existing isotopologs were detected.

For this reason, the rule set of the standard tracer approach required the presence and permissible intensity of at least two C_2_ extension unit isotopologs (X_+2_ and X_+4_) as well as the isotopologs X_+1_ and X_+3_ in between for a hit. In addition, the intensity ratios of the sum of X plus X_+2_ to X_+1_ and X_+2_ plus X_+4_ to X_+3_, respectively, had to be equal with a maximum permissible deviation of ±10%.

With the reversed tracer approach, more abundant signals for the X_–*n*_ isotopolog signals (X_–1_, X_–2,_...) were obtained compared to the standard tracer approach. Therefore, the rule set was extended by an additional pair of isotopologs corresponding to a third tracer-derived C_2_ extension unit (X_–5_ and X_–6_), further increasing the specificity.

### 3.2 Isotope Patterns Found for the Polyketides Aurofusarin and Sorbicillinol

**Figure 6:**
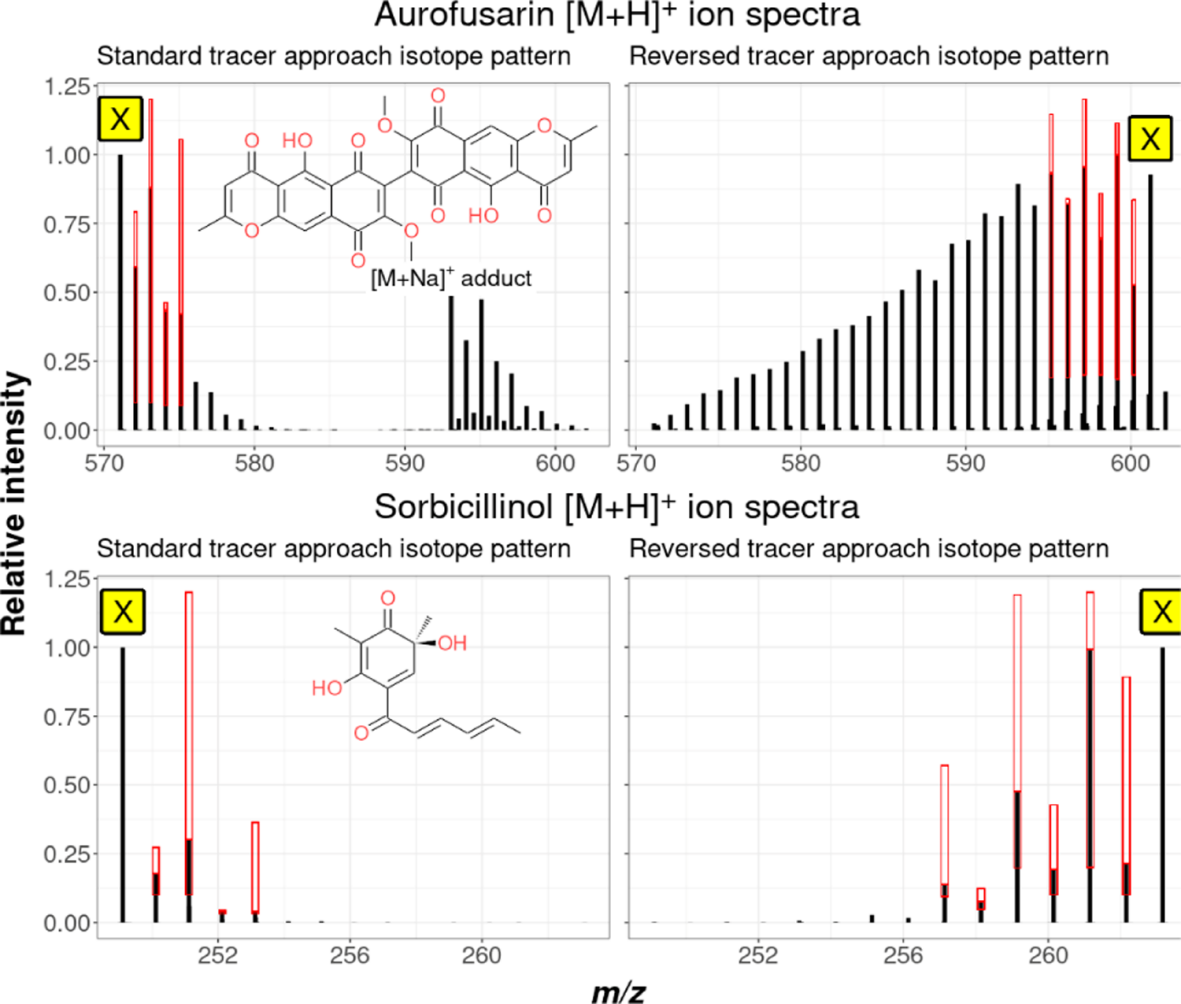
Spectra of the metabolites aurofusarin (*F. graminearum*) and sorbicillinol (*T. reesei*), actually found with CPExtract for both tracer approaches. Also shown in red boxes are the isotopologs defined in the rules that had to be present and their permitted intensity ranges.

The experimentally observed isotopolog patterns after data processing with CPExtract are exemplified with the two known polyketides aurofusarin for *F. graminearum* and sorbicillinol for *T. reesei*. The results are shown for both tracer approaches in Figure 6.

The spectrum of aurofusarin from the reversed tracer approach showed all 15 isotopolog signals corresponding to the incorporation of all 15 possible malonate tracer-derived C_2_ extension units into the carbon skeleton. In contrast, for aurofusarin in the standard tracer approach and for sorbicillinol in both approaches, only some of the theoretically possible isotopologs were sufficiently abundant for being detected.

### 3.3 Overall Pattern Shapes Found for the Polyketide Aurofusarin

**Figure 7:**
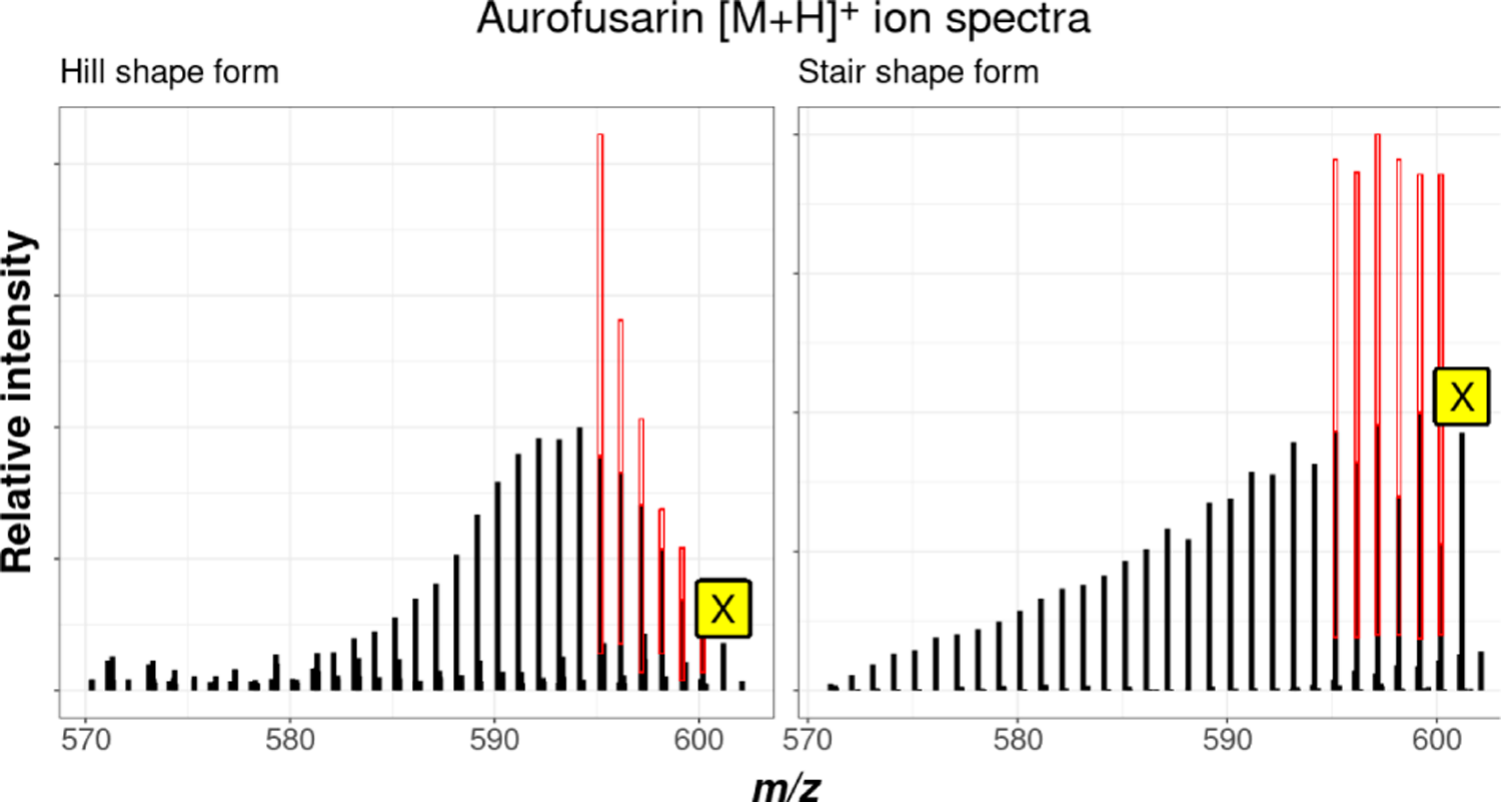
Different isotope pattern shapes of [M + H]^+^ isotopologs of *F. graminearum*-derived aurofusarin. A hill-shaped form was found in mycelium extracts (left), whereas a stair-shaped form was found in supernatant samples (right).

In both approaches, the intensity distribution does not necessarily have to follow the strict declining or increasing pattern shown above. Depending on the onset and time period of the metabolite synthesis during cultivation and the prevailing ratio of the added tracer to extension units formed by the fungus from the general carbon source, a different intensity profile can also appear. Moreover, the shape of the pattern also depends on whether a metabolite is possibly composed of two or more building blocks that were initially synthesized independently, as well as the cell compartment localization and time and rate of excretion of the biosynthesized product. Figure 7 illustrates, for e.g., the slightly differing isotope patterns obtained for the *F.* graminearum-derived polyketide aurofusarin (C_30_H_18_O_12_) in the mycelium extract and supernatant samples.

Consequently, the rules for the permissible intensity ranges for the X_±2_ isotopologs must allow adequate leeway for being able to detect all metabolite ions of the target substance class.

While the large tolerance windows needed for the initial pattern search can potentially compromise the filtering specificity and thus lead to false-positive search results, additional pattern shape characteristics can be used for further filtering of candidate ions.

Insufficient predictability of the overall shape of the pattern does not affect the respective intensities of the isotopologs located between two adjacent even-numbered isotopologs (e.g., for X_+1_ between X and X_+2_). As already explained above, the odd-numbered (X_±1_, X_±3_,...) isotopologs always result from the sum of the respective isotope fractions and the number of carbon atoms in the metabolite. Furthermore, and perfectly usable as a reliable selection criterion, the relative ratio of the intensity of these isotopologs to that of the respective two analogous neighboring isotopologs with a differing number of tracer-derived extension units must remain the same. Therefore, much smaller tolerable intensity intervals can be defined in the rules for the odd-numbered isotopologs without running the risk to miss target metabolite ions. To this end, the initially created candidate list can be refined by checking the constant relative intensities as described above to sort out the remaining false positives very reliably.

### 3.4 Global Screening of All Tracer-Derived Metabolite Ions

**Figure 8:**
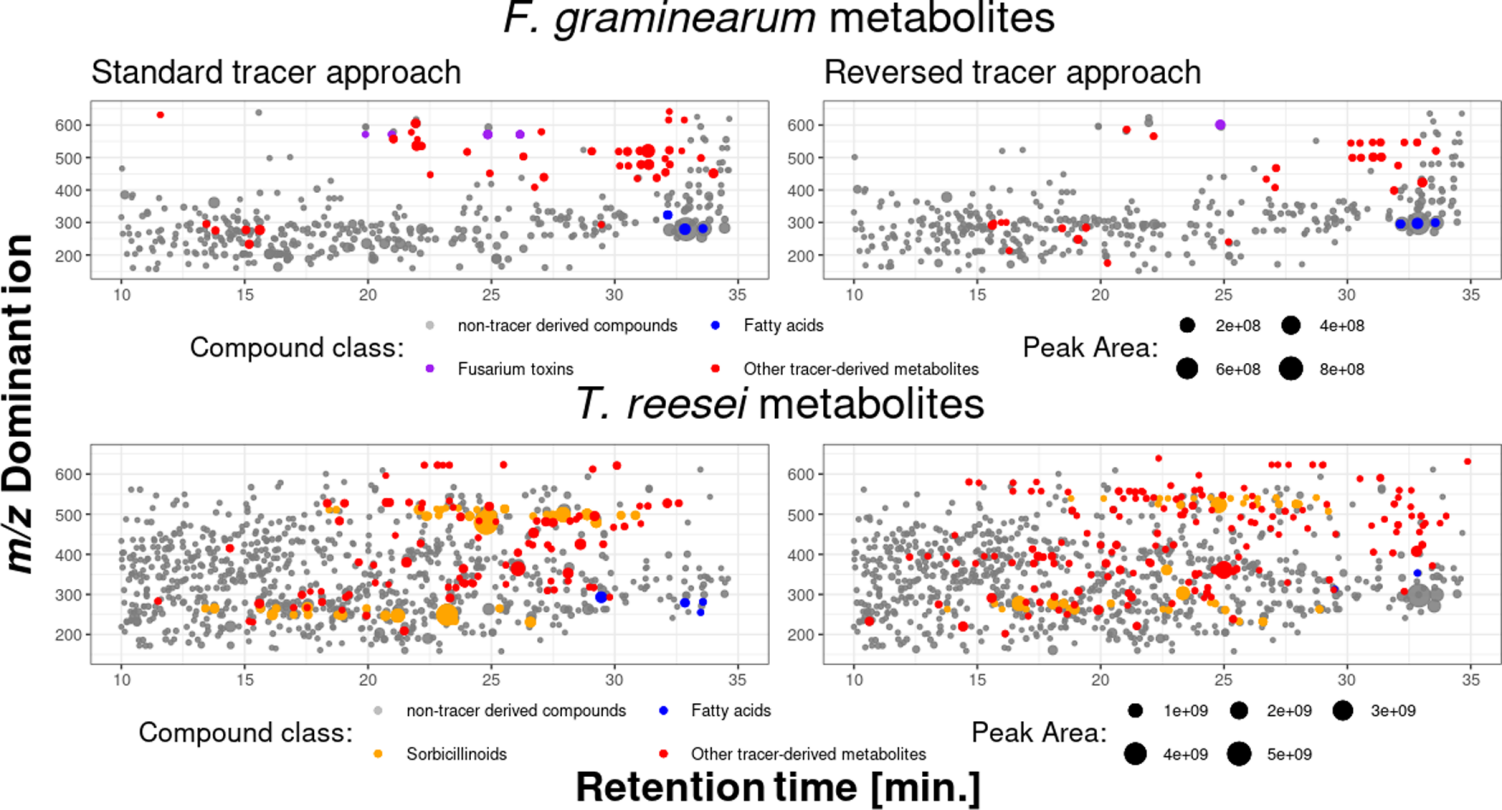
Mass-retention time plots of the dominant ion of each feature group from all truly fungal-derived metabolites found by MetExtract II software (shown in gray) in the sample data sets of *F. graminearum* and *T. reesei* (all three genotypes) culture samples. Metabolites detected by CPExtract are shown in purple (known *F. graminearum* metabolites), yellow (known *T. reesei* metabolites), blue (fatty acids), or red (unknown, potential novel tracer-derived metabolites), illustrating the comparability of both tracer approaches and the filter effect of the targeted search for tracer-derived metabolites.

The big advantage of filtering MS features according to certain metabolite classes by CPExtract using specific isotope patterns derived from the corresponding tracer can clearly be seen in Figure 8. Likewise, the comparability of the two complementary tracer approaches is illustrated.

In the *F. graminearum* data set, the MetExtract II evaluation of the global labeling approach resulted in a total number of 621 metabolites found both in the supernatant and mycelium extract samples of the wild-type strain PH-I that were actually produced by the fungus. The CPExtract algorithm detected a subset of tracer-derived metabolites from this set, comprising 39 metabolites for the standard tracer approach and 54 metabolites for the reversed tracer approach. In total, 46 of these metabolites could not be clearly identified with reference standards and thus represent possible novel metabolites.

This clearly demonstrates that the filter effect of the tracer approach combined with the automated CPExtract data evaluation on the targeted metabolic pathway worked properly.

The importance of each rule in defining the isotopic pattern to be searched for became apparent as each rule was added step by step. When using the intensity rule only, 122 and 91 possible targets were detected in the data of the standard and reversed tracer approaches, respectively, including a large number of false positives. After adding the absence rules, many of these false positives were removed and 83 (standard approach) or 64 (reversed approach) possible targets remained. After adding the ratio rules, the remaining false positives were removed, leaving 54 and 39 found metabolites in the standard and reversed tracer approach samples, respectively.

In the *T. reesei* data set, consisting of the supernatant and mycelium extract samples of each of the three different strains, 1165 metabolites truly originating from the fungus were found by MetExtract II data evaluation. The CPExtract algorithm data processing yielded 167 tracer-derived metabolites for the standard tracer approach and 283 for the reversed tracer approach, respectively. In total, 270 metabolites could not be clearly identified with reference standards and thus represent possible novel metabolites. When solely using the presence rules, 464 (standard approach) and 1462 (reversed approach) possible targets were detected. After adding the absence rules 282 (standard approach) or 418 (reversed approach) possible targets remained. The additional application of the ratio rule then yielded the remaining targets whose isotope pattern matched the expectation.

**Figure 9:**
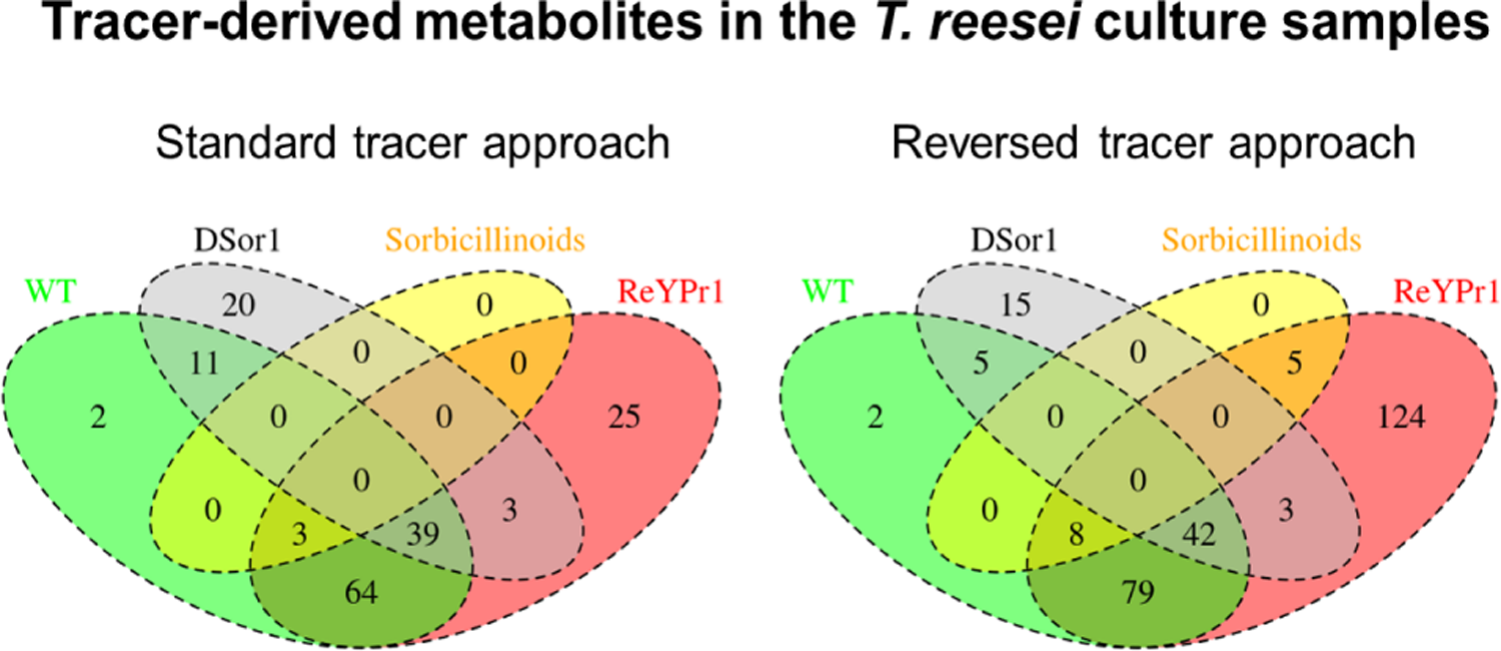
Venn diagrams of all metabolites found with both, the standard tracer and the reversed tracer approach in the investigated *T. reesei* strain culture samples (wild-type (WT) in green, Ypr1-overexpressing ReYpr1 strain in red, and Δ*sor1* deletion strain in gray). The annotated sorbicillinoids are shown in yellow.

The relatively high number of (malonate-derived) metabolites in the *T. reesei* samples can be explained, on the one hand, by the combination of three different fungal genotypes and is on the other hand presumably due to the high reactivity of the sorbicillinoids. Members of this class of polyketides are known to spontaneously react nonenzymatically into various other substances or reaction by-products. Figure 9 shows the all found feature groups detected in the *T. reesei* culture samples for both tracer approaches.

In the Venn diagrams, it can be seen that no metabolites annotated as sorbicillinoids were found in the ΔSor1 strain samples, which is as expected since this strain is deficient in the sorbicillinoid polyketide gene cluster and thus not able to produce any sorbicillinoids at all. Furthermore, additional sorbicillinoids (namely, putative bisorbicillinol, epoxysorbicollinol, sorbiquinol, bisvertinolone, dihydrobisvertinolone) were found exclusively in samples of the overexpressing ReYpr1 strain.

### 3.5 Selectivity of the CPExtract Algorithm

Furthermore, to test the selectivity of the CPExtract algorithm and the rule sets used, culture samples without the addition of the tracer compounds were measured and processed using the same settings. This was done for both the standard- (^12^C glucose) and reversed (^13^C_6_-glucose) approach. In these negative control samples, a low number of hits (false positives) were detected by CPExtract, demonstrating the high specificity of the presented approach. Surprisingly, only in some *T. reesei* negative control samples, the corresponding tracer-derived isotope patterns were detected. In total, seven corresponding tracer-derived patterns (different ones in each replicate) were detected in supernatant samples of the Ypr1-overexpressing ReYpr1 strain, and one tracer-derived pattern was detected in a single replicate of the WT supernatant samples. Manual inspection of the raw data files revealed that the false-positive signals were only detected immediately after the measurement of samples, which showed high signal intensities for exactly those ion species. Therefore, it was concluded that the false positives resulted from carryover into successive injections via the insufficiently rinsed injection needle of the HPLC system. Thus, the false-positive hits were not caused by a low selectivity of the CPExtract algorithm but correctly detected and annotated as polyketides (data not shown).

## 4 Concluding Remarks

Based on the exemplary search for metabolites of the malonate pathway in the culture samples of two different filamentous fungi using two complementary tracer approaches, it was possible to demonstrate the reliable function of the CPExtract algorithm and its universal and versatile applicability. Tracer-derived metabolites were detected by searching for very specific isotope patterns that do not occur naturally as a result of the random incorporation of tracer molecules during biosynthesis. The pattern to be searched for, was defined by means of rules describing it, which CPExtract then used for the search. Through the detection of unknown metabolites, specifically from the substance group of interest, in addition to the known and expected metabolites of the respective fungus, it is evident that CPExtract software can also benefit the search of various other putatively novel or even completely unknown metabolites. Especially the timely but still demanding topic of natural product discovery is a promising field of application of CPExtract. As exemplified in this article, the reliable filtering of the MS data for the fatty acids and polyketides searched for, enables a drastic reduction in the complexity of the global metabolite profiles. This can help to greatly simplify manual data curation that is subsequently required.

CPExtract also shows great potential for other natural product classes such as nonribosomal peptides or terpenoids when being combined with other tracer compounds such as amino acids or mevalonate. Another interesting option is to screen for functionalization of natural products like, for example, methylations using l-methionine-(methyl-^13^C). Finally, the metabolic fate of (labeled) potentially toxic xenobiotics can be probed by CPExtract either with the standard or reverse approach.

We anticipate that the presented tracer approach in combination with the flexible and easy-to-use CPExtract software will be of interest to the metabolomics community working in related fields of research.

CPExtract is available free of charge for noncommercial use at https://metabolomics-ifa.boku.ac.at/CPExtract. The source code is available at https://github.com/chrboku/CPExtract. Likewise, the LC-HRMS data can be obtained from https://metabolomics-ifa.boku.ac.at/CPExtract/#datasets.

## Supporting Information Available

One section with additional text and three figures describing the SIA-tracer workflow; cultivation; sample preparation and measurement in detail; and showing screenshots of CPExtract software (PDF)
